# Successful hemostasis and resection of a bleeding gastric polyp by endoscopic banding ligation in a uremic patient taking antiplatelet agent

**DOI:** 10.1186/s40064-016-3499-0

**Published:** 2016-10-18

**Authors:** Ping-I Hsu, Kwok-Hung Lai, Feng-Woei Tsay, Jin-Shiung Cheng, E-Ming Wang, Rong-Jer Lai, Tsair-Fwu Lee

**Affiliations:** 1 Division of Gastroenterology, Department of Internal Medicine, Kaohsiung Veterans General Hospital, National Yang-Ming University, Kaohsiung, Taiwan, ROC; 2 Department of Mechanical Engineering, National Kaohsiung University of Applied Science, Kaohsiung, Taiwan, ROC; 3 Medical Physics and Informatics Laboratory of Electronics Engineering, National Kaohsiung University of Applied Sciences, Kaohsiung, 80778 Taiwan, ROC; 4 Graduate Institute of Clinical Medicine, Kaohsiung Medical University, Kaohsiung, 807 Taiwan, ROC

**Keywords:** Banding ligation, Endoscopy, Bleeding polyp, Anti-platelet agent

## Abstract

The modalities to treat bleeding polyps include electrocautery snare polypectomy, adrenaline injection, clipping, argon plasma coagulation and surgery. We hereby describe an endoscopic banding ligation method for the management of bleeding gastric polyp in a patient receiving antiplatelet therapy. A 66-year-old man presented with a five month-history of intermittent tarry stool passage, nausea and fatigue. He had a past history of peripheral arterial occlusive disease and non-insulin dependent diabetes mellitus with end stage renal disease, and regularly took antiplatelet agent (ticlopidine 100 mg thrice daily) for cardiovascular prophylaxis. On examination, the patient was grossly pale, ill in appearance, with a pulse of 110/min and blood pressure of 108/76 mmHg. Laboratory examination revealed hemoglobin of 7.8 g/dl. Endoscopic examination revealed a bleeding sessile polyp over the posterior wall of the antrum. Endoscopic banding ligation was carried out by a pneumoactivated esophageal variceal ligation device set. Bleeding stopped immediately following the procedure, and the patient recovered uneventfully. It is suggested that endoscopic banding ligation is a safe and effective technique for the treatment of bleeding gastrointestinal polyps in patients receiving antiplatelet therapy.

## Introduction

Polyp or tumor bleeding is an uncommon cause of upper gastrointestinal hemorrhage (Van Leerdam 2008; Suo et al. 2011; Lakhwani et al. 2000). The modalities to treat bleeding polyps or tumors include electrocautery snare polypectomy, adrenaline injection, clipping, argon plasma coagulation and surgery (Hirasaki et al. 2009; Al-Haddad et al. 2007; Malik et al. 2011; Euanorasetr and Sornmayura 2010; Shah et al. 2015). Although electrocautery snare polypectomy is a common method to treat non-bleeding gastrointestinal polyps, hemorrhage is a serious and most common complication of electrocautery snare polypectomy with an incidence ranging from 1.0 to 7.2 % in prospective studies (Lanza et al. 1981; Muehldorfer et al. 2002; Chin-Lin Perng et al. 2001; Ji et al. 2014). This endoscopic method therefore carries a potential risk of inducing bleeding in the treatment of bleeding lesions. Furthermore, use of antiplatelet agent increases the risk of electrocautery snare polypectomy-related bleeding. A meta-analysis of five observational studies concerning clopidogrel use with polypectomy compared 574 patients who continued clopidogrel therapy before polypectomy with 6169 control patients. A significantly increased risk of delayed post- polypectomy bleeding with relative risk of 4.7 was demonstrated (Malik et al. 2011; Kida et al. 2012). In addition to electrocautery snare polypectomy, local injection, clipping and thermocoagulation have been employed in the management of bleeding gastrointestinal tumors. Kida et al. (2012) reported a case of a small-bowel lymphangioma with bleeding that were not amenable to adrenaline injection and hemoclipping and finally successfully treated by argon plasma coagulation.

Endoscopic ligation using suction equipment and rubber bands has been widely used in the management of bleeding esophageal varices (Cipolletta et al. 1998; Wang et al. 2014). The varices are automatically eradicated through the use of ligation. In this case report, we applied endoscopic banding ligation (EBL) to treat a bleeding gastric polyp in a uremic patient who took antiplatelet agent for secondary prevention of cardiovascular disease. To our knowledge, this is the first case with bleeding gastric polyp treated by EBL.

## Case report

**Fig. 1:**
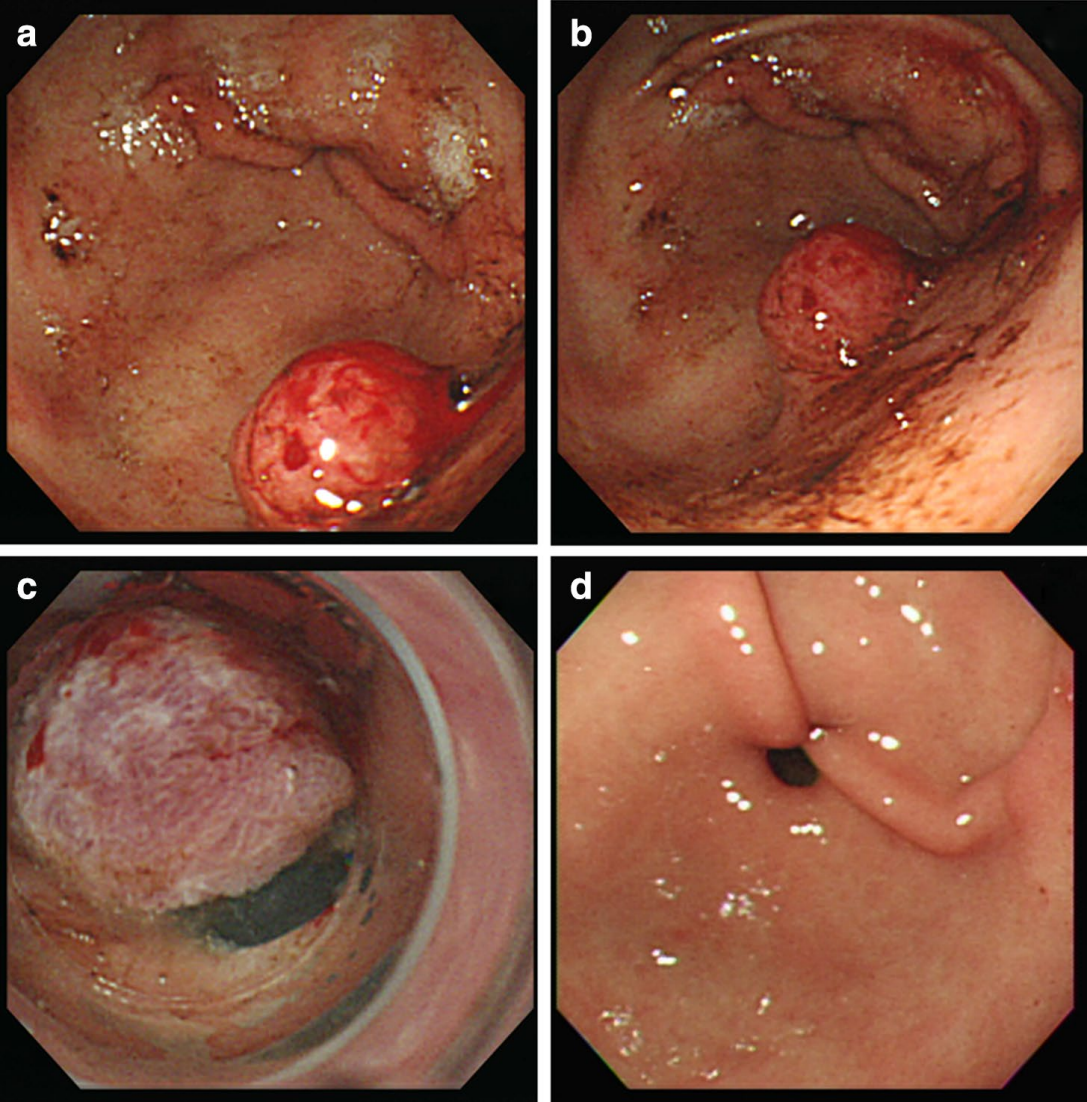
Endoscopic banding ligation (EBL) for a bleeding gastric polyp. **a** A bleeding polyp over the posterior wall of the antrum. **b** The polyp was lifted following injection of 3 ml of distilled water into its base. **c** Bleeding stopped following EBL, and the polyp developed cyanotic change. **d** A follow-up endoscopy revealed that both the ligated polyp and rubber band had disappeared

**Fig. 2:**
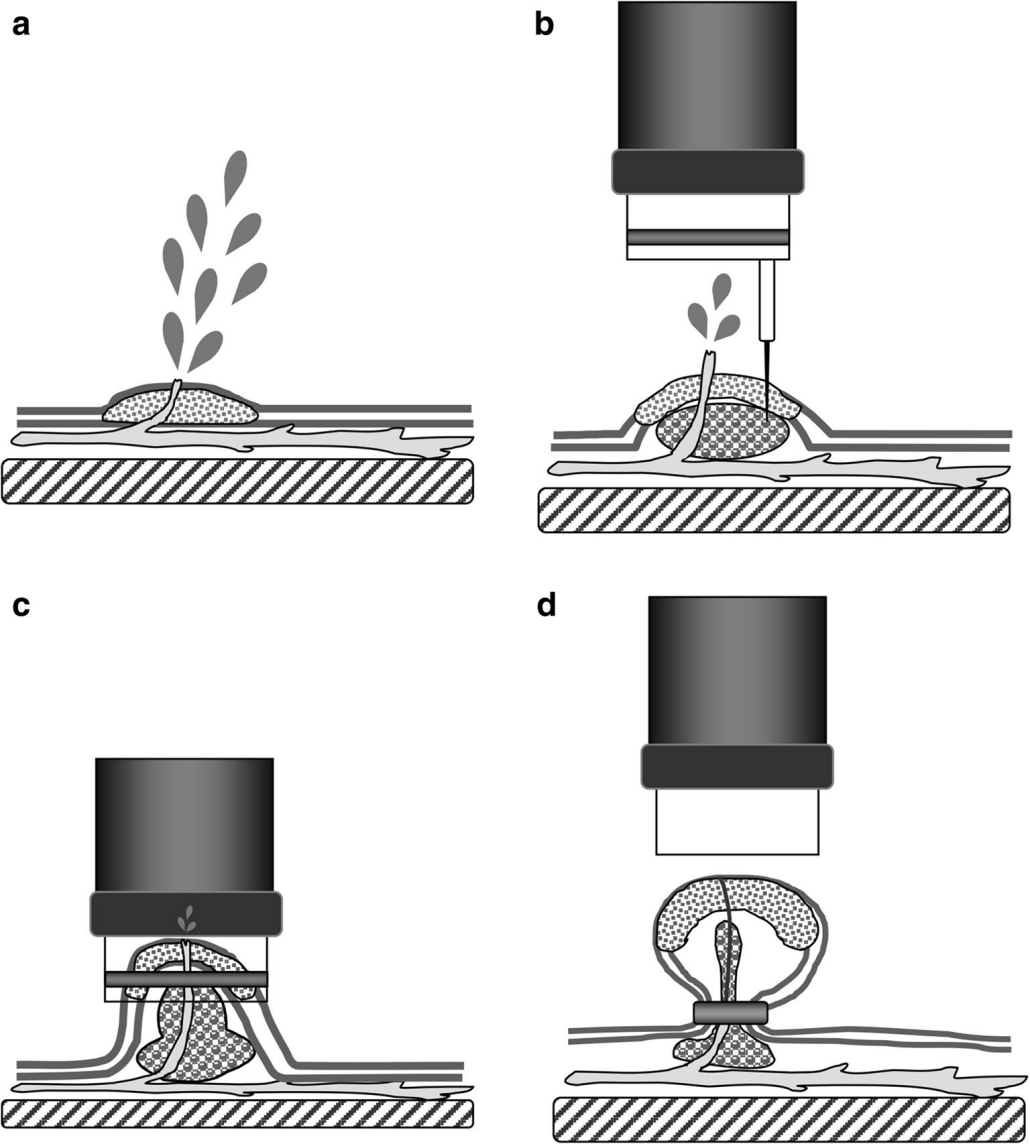
**a** A bleeding polyp. **b** Distilled water was injected locally into the submucosa adjacent to the bleeding polyp to lift the lesion from the muscle layer. **c** The raised lesion was then aspirated into the hood. **d** The polyp was ligated by a rubber band and bleeding stopped immediately

A 66-year-old man presented with a five month-history of intermittent tarry stool passage, nausea and fatigue. He had a past history of peripheral arterial occlusive disease, coronary artery disease and non-insulin dependent diabetes mellitus with end stage renal disease for years. In order to prevent thrombotic events, he took antiplatelet agent (ticlopidine 100 mg thrice daily) regularly. On examination, the patient was grossly pale and ill in appearance with a pulse of 110/min and blood pressure of 108/76 mmHg. Digital examination documented the presence of tarry stool. Laboratory examination revealed hemoglobin of 7.8 g/dl, total leukocyte count of 6140/mm^3^ and platelet count of 200,000/mm^3^. The patient was subjected to upper gastrointestinal endoscopy, which revealed a bleeding sessile polyp in the posterior wall of the antrum (Fig. 1a). We performed EBL with a GIF XQ200 endoscope (Olympus Optical Co., Tokyo, Japan). The distal end of the scope was set a transparent hood equipped with a pneumoactivated esophageal variceal ligation (EVL) device set (Sumitomo Bakelite Co., Tokyo, Japan). It was composed of an air feeding tube, a sliding tube and a rubber band. Hyoscine-N-butylbromide 20 mg was given intramuscularly as premedication 5 min before the procedure. We placed an overtube outside the scope, and inserted the endoscope first. The overtube was then inserted through the endoscope. In order to lift the bleeding polyp, 3 ml of distilled water was injected into its base (Figs. 1b, 2b). We removed the scope later, and assembled the pneumoactivated EVL device set on the scope, which was then reinserted via overtube. We aspirated the raised polyp into the hood (Fig. 2c) and ligated it by rubber band later. Bleeding stopped immediately following banding procedure. The lesion became cyanotic approximately 4 min later (Figs. 1c, 2d). Following the procedure, pantoprazole 40 mg daily was administered orally for 4 weeks. He consumed a liquid meal for 24 h, and then took a regular diet. Ticlopidine was hold for 7 days, and restarted later. The patient recovered uneventfully, and no tarry stool recurred 2 days following EBL. A follow-up endoscopy 3 months later revealed that both the ligated polyp and the rubber band had vanished (Fig. 1d).

## Discussion

Antiplatelet therapy and uremic toxins can impair platelet function and hinder hemostasis during gastrointestinal bleeding. In this case report, we applied EBL method to treat a bleeding gastric polyp in a uremic patient who took ticlopidine for cardiovascular prophylaxis. Hemostasis was successfully achieved by banding ligation. Follow-up endoscopy showed that both the ligated polyp and the rubber band had disappeared spontaneously. The findings suggest that EBL is a safe and effective technique for the treatment of bleeding gastrointestinnal polyps in patients with bleeding tendency.

Our banding ligation method can be employed for both bleeding sessile or pedunculated gastric polyps. Sessile polyps are often difficult to be captured by traditional electrocautery polypectomy. Although endoscopic mucosal resection with cap-fitted panendoscopy may capture the lesions by suction, bleeding is a possible serious complication following the procedure (Inoue et al. 1993), especially in patients with bleeding tendency. In this situation, banding ligation of the lesions is possibly a good choice because it is easy to aspirate the polyps by suction, and the avoidance of resection procedure might decrease the risk of bleeding. In this reported case, 3 ml of distilled water was injected into the base of the bleeding polyp to lift the lesion off the muscle layer. Additionally, submucosal injection before banding ligation could compress the vessels in the submucosal layer and was possibly beneficial for preventing bleeding.

Conventional snare polypectomy encounters difficulties in effectively and efficiently controlling lesions located in the lesser curvature side, posterior wall and cardia of the stomach. Our previous study (Lo et al. 2003) showed that it was easily to capture a lesion into the hood of EVL, even the lesion was situated tangentially. Therefore, banding ligation method also can be applied for the treatment of bleeding gastric polyps located in some difficult approach areas.

In this reported case, the polyp immediately stopped bleeding following strangulation with the rubber band, and developed cyanotic change approximately 4 min later. It disappeared at follow-up endoscopy. The finding suggested that the ligated polyp would develop avascular necrosis following banding ligation. Therefore, our banding ligation could not only stop bleeding from polyp but also could bloodlessly transect the polyp. Since the bleeding polyp was not biopsied or resected following ligation, its nature was unknown. However, we have taken biopsies to assess the histology of some non-bleeding gastric polyps after banding ligation recently. No polyps bleeding occurred following biopsies because blood supply of those polyps was blocked by banding ligation. Therefore, it is possibly safe to take biopsy for bleeding polyps following banding ligation in patients with bleeding tendency.

The indication of banding ligation of gastrointestinal polyps include (1) treatment for bleeding polyps in patients with bleeding tendency, and (2) treatment for sessile or pedunculated hyperplastic polyps less than 1 cm. Because the distal attached hood of the pneumoactivated ligation device is 1 cm in diameter, it is difficult to capture a polyp greater than 1.5 cm by banding ligation. If the size of this polyp is greater than 1.5 cm, endoscopic detachable snare ligation method can be employed to ligate the polyps (Hsu et al. 2001). However, banding ligation technique is not suitable for treating a non-bleeding gastric adenoma, which carries a risk of carcinomatous conversion.

## Conclusions

Endoscopic banding ligation is a safe and effective technique for the treatment of bleeding gastrointestinal polyps in patients receiving antiplatelet therapy.
